# A Global Approach for Quantitative Super Resolution and Electron Microscopy on Cryo and Epoxy Sections Using Self-labeling Protein Tags

**DOI:** 10.1038/s41598-017-00033-x

**Published:** 2017-02-02

**Authors:** Andreas Müller, Martin Neukam, Anna Ivanova, Anke Sönmez, Carla Münster, Susanne Kretschmar, Yannis Kalaidzidis, Thomas Kurth, Jean-Marc Verbavatz, Michele Solimena

**Affiliations:** 1 Molecular Diabetology, University Hospital and Faculty of Medicine Carl Gustav Carus, TU Dresden, Dresden Germany; 2 Paul Langerhans Institute Dresden (PLID) of the Helmholtz Center Munich at the University Hospital Carl Gustav Carus and Faculty of Medicine of the TU Dresden, Dresden, Germany; 3 Center for Regenerative Therapies Dresden (CRTD), TU Dresden, Dresden Germany; 4 Biotechnology Center of the TU Dresden (BIOTEC), Dresden, Germany; 5 Max Planck Institute of Molecular Cell Biology and Genetics (MPI-CBG), Dresden, Germany; 6 Faculty of Bioengineering and Bioinformatics, Moscow State University, Moscow, Russia; 7 Institut Jacques Monod, Université Paris Diderot, Paris, France

## Abstract

Correlative light and electron microscopy (CLEM) is a powerful approach to investigate the molecular ultrastructure of labeled cell compartments. However, quantitative CLEM studies are rare, mainly due to small sample sizes and the sensitivity of fluorescent proteins to strong fixatives and contrasting reagents for EM. Here, we show that fusion of a self-labeling protein to insulin allows for the quantification of age-distinct insulin granule pools in pancreatic beta cells by a combination of super resolution and transmission electron microscopy on Tokuyasu cryosections. In contrast to fluorescent proteins like GFP organic dyes covalently bound to self-labeling proteins retain their fluorescence also in epoxy resin following high pressure freezing and freeze substitution, or remarkably even after strong chemical fixation. This enables for the assessment of age-defined granule morphology and degradation. Finally, we demonstrate that this CLEM protocol is highly versatile, being suitable for single and dual fluorescent labeling and detection of different proteins with optimal ultrastructure preservation and contrast.

## Introduction

Fluorescence light microscopy (FLM) is one of the most common methods in cell biology and many different fluorescent markers can be used to visualize cellular components, protein distribution, signaling events or biochemical reactions in living cells. However, the resolution of FLM is limited by diffraction^1^. Moreover, only labeled structures can be imaged, whereas unlabeled structures in the vicinity, the so-called reference space, remain invisible. Transmission electron microscopy (TEM), on the other hand, reveals subcellular details of both labeled and unlabeled structures, but it is limited to fixed samples and labeling options are restricted to a handful of particulate markers. Correlative light and electron microscopy (CLEM) enables the detection of fluorescently labeled proteins in electron microscopy images. There are several ways to perform correlative experiments combining different methods for FLM microscopy, various embedding and sectioning techniques, and different EM techniques^2,3^. Generally, a CLEM method is defined to be a pre-embedding or post-embedding technique based on when FLM is performed. Pre-embedding protocols usually involve live-cell FLM and subsequently tracking of the objects in sections of the embedded sample^4–8^, while post-embedding protocols rely on cryotechniques or special embedding media for retention of the initial fluorescent signal even after sample processing for EM^9–20^. While pre-embedding CLEM is focused on single events and a small sample number, post-embedding CLEM allows for screening higher numbers of cells, with detection and correlation of several events. Traditionally, the protein of interest is detected either by immunofluorescence and immunogold labelings with antibodies or tagged with a fluorescent protein (FP). Notably, post-embedding CLEM by preservation of fluorescence in epoxy resins, the most commonly used resins in EM, could never be shown before, since FP-based probes are susceptible to strong fixation and photobleaching. Hence, CLEM of resin embedded samples relies on either photoconversion of fluorescence or the use of methacrylate resins. Moreover, immunolabeling or FPs are only suitable for the detection of a protein population as a whole. On the other hand, recently developed self-labeling proteins such as SNAP- and CLIP-tag (New England Biolabs) can be used similarly to FPs, except for the need of an additional labeling step with highly photostable organic fluorescent substrates^21^. The availability of non-fluorescent substrates further allows for “pulse-chase” experiments, i.e. the labeling of pools of the target protein generated at different timepoints. Self-labeling protein-tags have been used for live-cell imaging^22–24^ and also for post-embedding CLEM with metacrylate resin^20^ and pre-embedding CLEM by photo-oxidation of a fluorescent substrate^25^. Recently, we showed that a fusion construct between human insulin and SNAP (hIns-SNAP), is a reliable reporter for fluorescent labeling of age-distinct insulin secretory granules (SGs)^26,27^. With this technique age-distinct pools of insulin SGs can be independently labeled through sequential incubations with fluorescent and non-fluorescent SNAP substrates.

Here we label insulin SGs of different age in beta cells of pancreatic islets isolated from SOFIA (Study of insulin aging) mice, in which an *insulin2-SNAP* allele had been knocked-in into the *Ins2 locus*. We preserve the initial fluorescence of SNAP-substrates TMR-Star and 505-Star in ultrathin frozen Tokuyasu sections. We examine these specimens with structured illumination microscopy (SIM) and are able to correlate SIM images to their corresponding EM images with high precision. This allows for the investigation of age-dependent insulin SG pools at the ultrastructural level. We show that the number of newly-generated insulin SGs reduces starting at an age of 2.7 days, with 60% of labeled insulin being degraded in multi-granular bodies (MGBs) after 5 days. Furthermore, we develop protocols for the preservation of SNAP- and CLIP-based fluorescence in epoxy resin. This allows for the morphometrical evaluation of age-defined insulin SGs and investigation of their degradation by autophagy. Moreover, we demonstrate that tagging of several cytosolic, membrane-associated, nuclear and cytoskeletal proteins with either SNAP or CLIP followed by labeling with fluorescent substrates also enables their detection in Epon epoxy resin sections by FLM after high pressure freezing (HPF) and freeze-substitution (FS) and even after chemical fixation. We thereby demonstrate the versatility of our approach, which can be applied to a variety of other proteins than insulin.

## Results

### Labeling Protocols for Self-labeling Protein Tags

**Figure 1:**
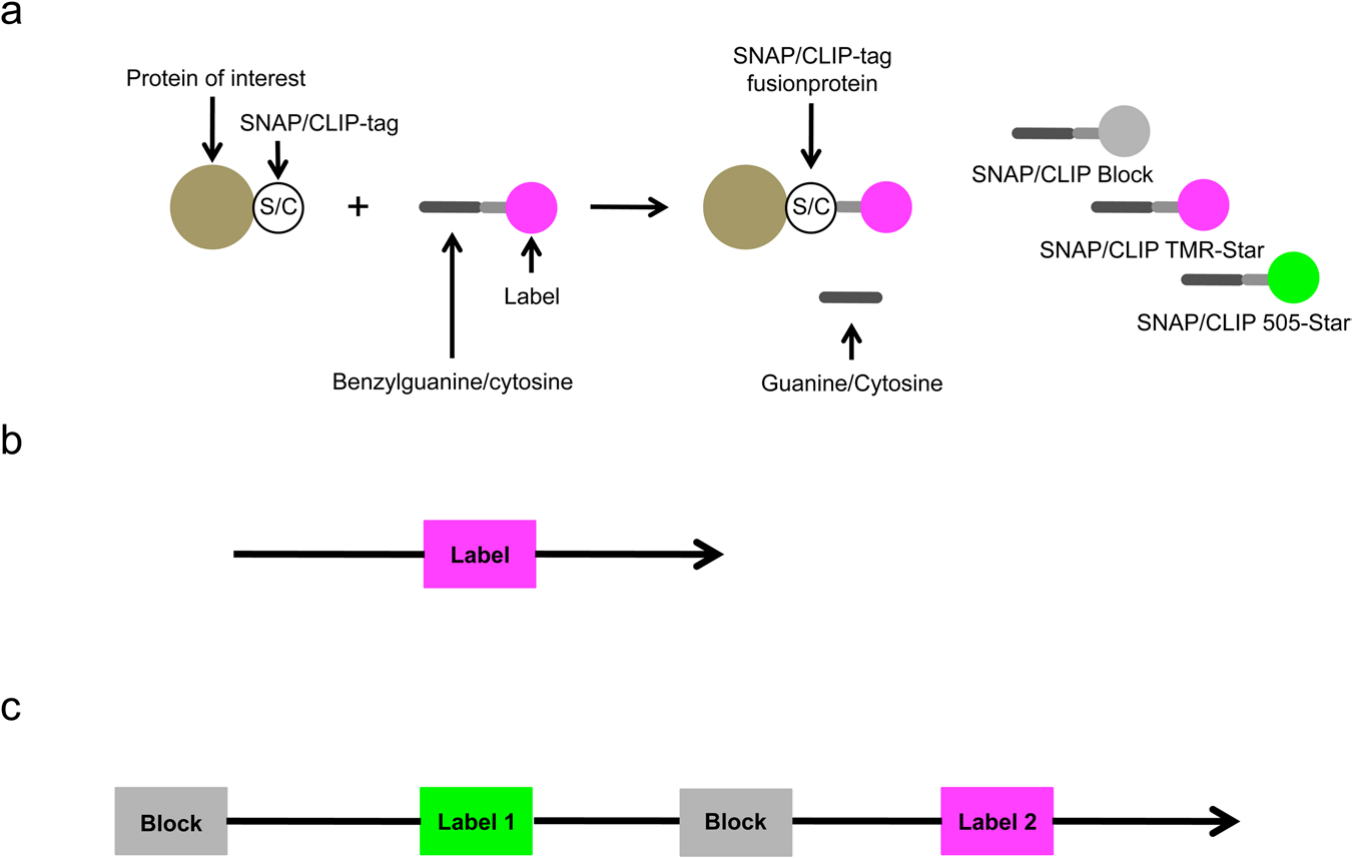
Principle of SNAP/CLIP-labeling and labeling protocols. (**a**) SNAP- and CLIP-tag fused to the protein of interest bind benzylguanine- or benzylcytosine-substrates, respectively. There are various cell-permeable fluorescent substrates and a non-fluorescent substrate available. (**b**) For labeling of the whole fusion protein pool cells are incubated with a fluorescent substrate, washed and fixed/imaged. (**c**) For age-dependent labelings cells are first incubated with the non-fluorescent blocker followed by labeling of the newly-synthesized fusion protein with a fluorescent substrate. To omit continuous labeling with the first substrate additional blocking/labeling steps can be added.

Self-labeling protein tags require a labeling step with fluorescent substrates in order to perform FLM imaging. There are fluorescent substrates with different wavelengths available, as well as a non-fluorescent substrate that can be used as a blocker (Fig. 1a). If the labeling of the whole pool of the tagged protein was desired, we applied a simple labeling protocol that included the incubation of the cells with one fluorescent substrate (Fig. 1b). Saturation of pre-existing tagged proteins in the cells with the blocker was instead required to achieve a starting point for age-dependent labeling of the newly synthesized tagged proteins by addition of the fluorescent substrate. Depending on the desired age of the tagged protein to be imaged, cells were then kept in culture for a variable period of time. If necessary, a substrate with a different wavelength was added at a later time point to ensure the correct age-dependent labeling (Fig. 1c).

### Quantitative Super Resolution Light and Electron Microscopy of Age-Distinct Insulin Granules in Mouse Pancreatic Beta Cells

The preferential release of newly synthesized proteins has been shown for several secretory cells, mostly neurons^28–31^. It has also been known for several decades that pancreatic beta cells preferentially release newly synthesized insulin^32–35^. To investigate SG ageing in primary beta cells we created the SOFIA mouse with *insulin2-SNAP* knocked in into the *insulin2 locus* called SOFIA mouse. We could previously show that in this mouse model the insulin2-SNAP reporter is correctly targeted to insulin SGs and SNAP substrates specifically label these vesicles^26^.

**Figure 2:**
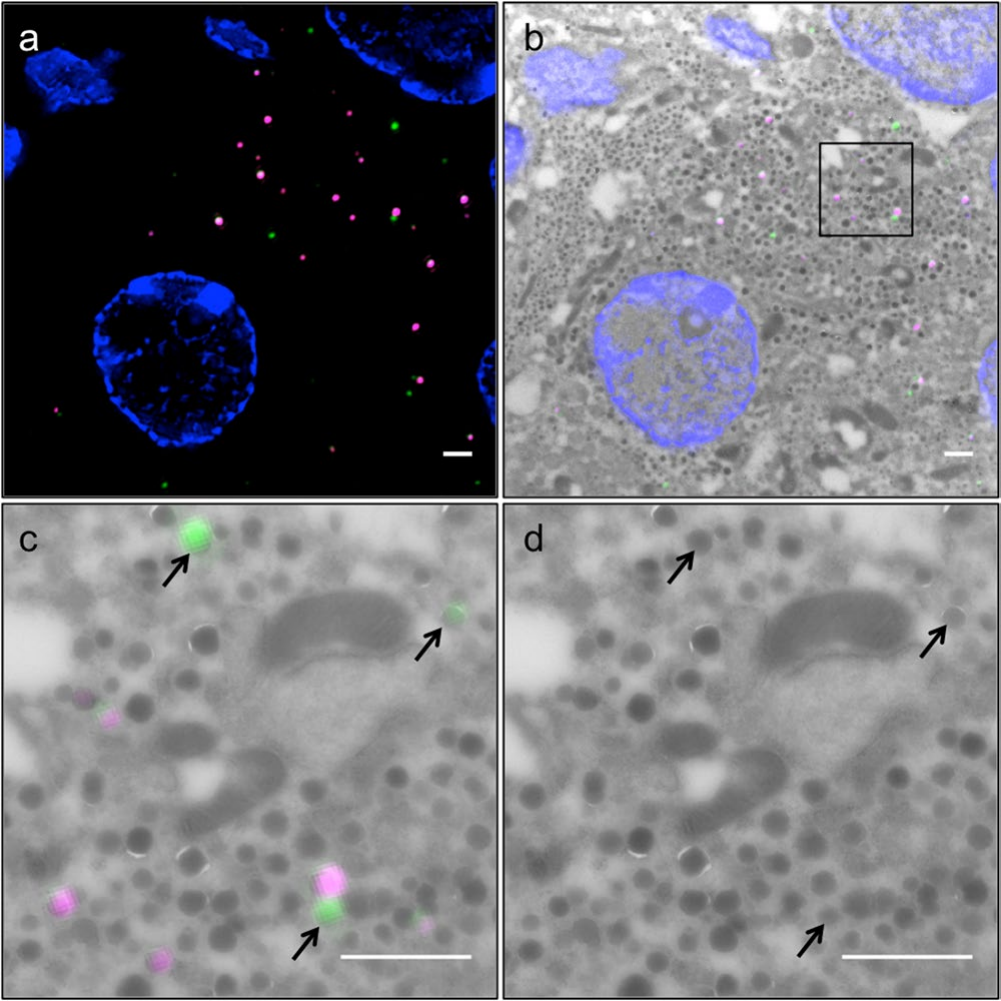
Age-dependent labeling of insulin SGs and super resolution CLEM. (**a**) SIM image of Tokuyasu section of SOFIA mouse beta cells labeled according to dual-labeling protocol with 505+ (green), 505+/TMR+ double labeled and TMR+ (magenta) SGs. (**b**) Overlay of SIM image with corresponding TEM image. (**c**) Detail from (**b**) 505+ and TMR+ SGs are precisely corresponding to their TEM counterparts. (**d**) TEM detail, arrowheads point to correlated 505+ SGs, Scale bars: 1 µm.

To investigate insulin SG ageing in primary SOFIA mouse beta cells we employed a post-embedding CLEM approach using Tokuyasu cryo-sections rather than live-cell imaging of primary beta cells. The latter approach would require the dispersion of isolated pancreatic islets into single cells, with possible alterations in the rates of insulin SG biogenesis and consumption upon exocytosis or intracellular degradation. Furthermore, the close contiguity of insulin SGs and their average diameter of 243 nm^36^, close to the diffraction limit of FLM, hamper the unequivocal identification of individual SGs. Ultrathin cryo-sections were therefore analyzed with structured illumination microscopy (SIM), which allows the resolution to be increased 2 fold relative to conventional FLM^37^. The SNAP-substrates 505-Star and TMR-Star used in this study could be well reconstructed and photo-bleaching leading to reconstruction artifacts was not detected (Fig. 2a), which was confirmed by checking indicators of reconstruction quality in the log-files and additionally assessing channel intensity profiles with the SIMcheck plugin for FIJI^38^ of representative images. Also, the integrity of the ultrastructure in Tokuyasu sections was sufficient for analysis (Fig. 2d). For the correlation of SIM and TEM images of Tokuyasu sections features of the nuclei present in both images were manually selected as landmarks using the Landmark function in AMIRA (FEI). The correlated images showed an almost perfect overlay of SIM and TEM images in their center and close to the landmarks (Fig. 2b,c). The mean shift between SGs in SIM and their EM counterparts was 51.3 ± 30.4 nm, which is below the average radius of an insulin SG of 121.5 ± 36.5 nm^36^ (Supplementary Figure S2). Ultimately, this method revealed precise correlation in the nm range and allowed for the reliable identification of labeled SGs within a large pool of unlabeled SGs sharing the same morphological features in TEM images.

**Figure 3:**
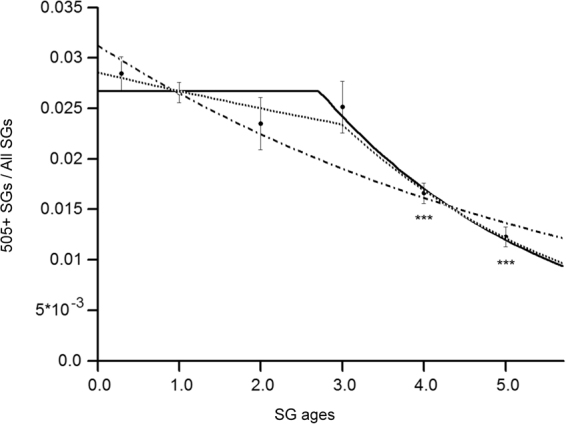
Quantification of age-defined insulin SGs in CLEM images. 505+ SGs/All SGs for SG ages of 5–8 h to 5 days. The ratio of labeled to all SGs remains relatively stable up to day 3 and shows significant reduction in day 4 and 5. Error bars indicate SEM. Level of significance is indicated with asterisks (n = 3). Solid line: model including lag-phase of no decay with duration Δ followed by an exponential decay (most probable). Dash-dotted line: model considering exponential decay of the number of labeled SGs. Dotted line: model including phase of slow decay with characteristic time τ_1_ for time Δ and fast decay with characteristic time τ_2_.

To achieve a correct time-resolved detection of SGs ranging in their age from several hours up to several days we applied a protocol involving sequential labelings of insulin SGs of SOFIA mouse beta cells with SNAP-Cell 505-Star as primary and SNAP-Cell TMR-Star as secondary fluorescent substrate, each preceded by a blocking step with the non-fluorescent SNAP substrate (Fig. 1c). As pancreatic islets are dense clusters of several hundred cells, the efficient removal of cell-permeable SNAP substrates is cumbersome, even with repeated washing steps. Hence, a protocol with only one substrate would not be accurate for an age-dependent labeling because persistency of the fluorescent probe could lead to the marking of SGs generated over a period of time longer than the desired one. This hurdle was overcome with a double labeling protocol, which resulted in three fluorescent SG pools within each labeled beta cell: one positive for SNAP-Cell 505-Star (505+), one positive for SNAP-Cell TMR-Star and one positive for both substrates (Fig. 2a). The two latter pools were discarded from the analysis, since the age of those SGs could not be reliably determined. Analysis of 505+ SGs in double labeled SOFIA beta cells revealed a constant percentage until the SG age of 3 days, ranging from 2.8 ± 0.16% to 2.5 ± 0.26%, followed by a reduction at day 4 and 5 to 1.7 ± 0.10% and 1.2 ± 0.10%, respectively (Fig. 3). Statistical analysis by ANOVA showed a significant difference between the age-dependent SG pools (F = 13.03, p__value_ < 0.01). To assess differences between single groups in detail *post hoc* Student t-test were applied (Supplementary Table 1). These t-test showed no differences among the first four age groups (5–8-hour-, 1-day-, 2-day and 3-day-old SGs), which were therefore treated as one large group for comparative t-tests with 4- and 5-day-old SGs. Specifically, there were significantly more 5–8-hour-, 1-day-, 2-day- and 3-day-old 505+ SGs compared to 4- and 5-day-old SGs (p__value_ = 0.0009 and p__value_ = 2.9·10^−5^, respectively), (Fig. 3, marked by asterisks).

Next, we estimated the parameters of the age-dependent behavior of insulin SGs in mouse beta cells exposed to 5.5 mM glucose by fitting 3 mathematical models of decay of labeled SGs to the data. The first model considered the exponential decay of the number of labeled SGs (Fig. 3, dash-dotted line, equation (1)). The second model included a lag-phase of no decay with duration Δ that was followed by an exponential decay with characteristic time τ (Fig. 3, solid line, equation (2)). The third model included a phase of slow decay with characteristic time τ_1_ for time Δ and fast decay with characteristic time τ_2_ (Fig. 3. dotted line, equation (3)). The Bayesian model probability estimation with uniform prior^39^ revealed that the second model was ~5 fold more probable than the first (simplest) model and ~1,000 fold more probable than third (most complex) model (see Methods for equations and details). Although the third model had the best fit to the experimental data, the improvement of the fitting quality was not sufficient to justify the choice of this more complex model. Therefore, following the Occam razor principle the second model was chosen. This model gave an estimate of the ageing lag phase duration Δ = 2.7 ± 0.4 days and the decay exponent τ = 2.8 ± 0.8 days. Hence, the effective half-life of SGs in mouse beta cells cultured in 5.5 mM glucose is Δ + ln(2)·τ = 4.6 days.

### Ageing of Insulin Granules Correlates with their Intracellular Degradation

**Figure 4:**
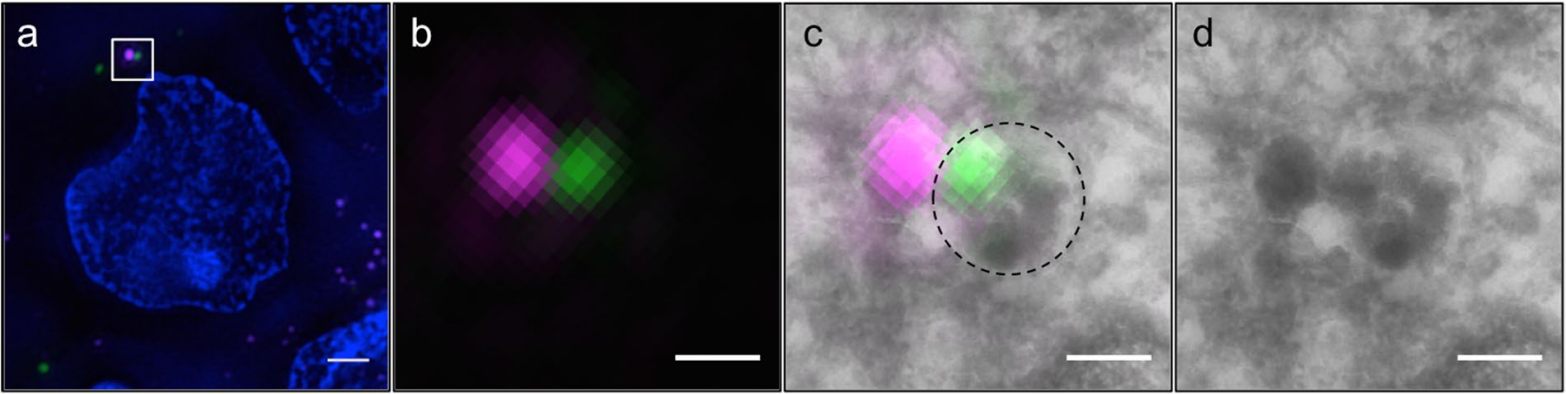
Degradation of old insulin SGs in MGBs. (**a**) SIM image SIM image of Tokuyasu section of SOFIA mouse beta cells labeled according to dual-labeling protocol with 3-day-old 505+ (green) and TMR+ (magenta) SGs. (**b**) Detail of boxed area with 505+ (green) and TMR+ (magenta) SG. SIM allows for the distinction of two adjacent SGs labeled with different fluorophores. (**c**) CLEM overlay revealing a 505+ SG within a MGB while a TMR+ SG is adjacent to the MGB (MGB is marked with a dashed line). (**d**) Corresponding TEM image of a MGB with a single SG in close proximity to its membrane. Scale bars: 1 µm.

Visual inspection revealed the presence of 505+ electrondense particles in MGBs, which are the sites of intracellular insulin degradation^40,41^ (Fig. 4). In particular, the number of 505+ SGs within MGBs were increased over time from 0–2 cases for 5–8 hrs-old to 2 days-old SGs to 3–6 cases for 3 to 5 days-old SGs (Supplementary Table 2). Since these data were not normally distributed, we tested their statistical significance using a Bayesian approach (Supplementary Methods). This analysis revealed that MGBs containing older (4- and 5-day-old) 505+ insulin SGs were significantly more compared than those containing younger 505+ insulin SGs (5–8 h to 2 days) (Supplementary Figure 3, Supplementary Table 3). Moreover, MGBs containing 3-day-old 505+ SGs were significantly more that those positive for 5-8-h-old 505+ SGs. Hence, an age of 3 days marks the turning point for increased probability of a SG to be degraded. The abrupt drop in the number of 4- and 5-day-old SGs relative to younger SGs, which are preferentially released, combined with the increased presence of older SGs in 505+ MGBs suggests that in our experimental conditions, i.e. in isolated islets exposed to 5.5 mM glucose, the consumption of SGs older than 2.7 days is mainly accounted by their intracellular degradation.

### Reassessment of Mouse Insulin SG Size by SNAP/CLIP-Based CLEM in Epoxy Resin

**Figure 5:**
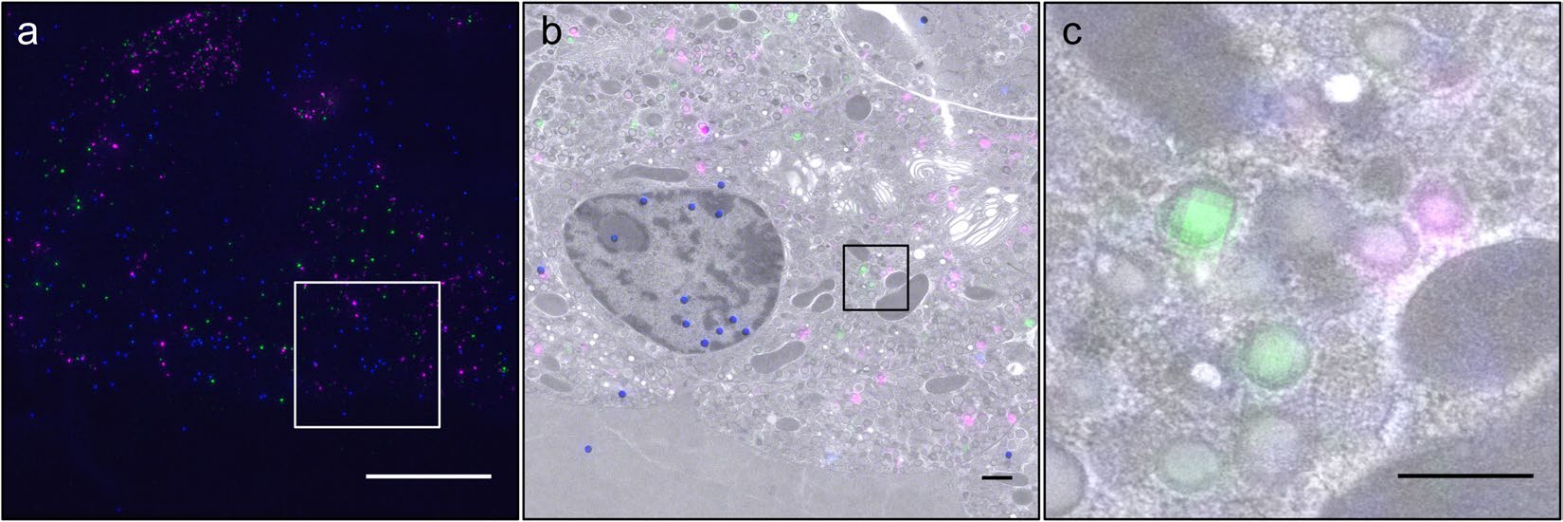
Super resolution CLEM in Epon sections. (**a**) SIM image of Epon section of SOFIA mouse beta cells labeled with 505-Star (green) and TMR-Star (magenta) fixed by HPF followed by FS. As fiducial markers fluorescent beads (blue) were added to the section prior to imaging. Punctate SGs can be unequivocally distinguished by SIM. (**b**) CLEM image of boxed area in (**a**). (**c**) CLEM detail of boxed area in (**c**) showing perfect overlay of 505+ and TMR+ SGs. Scale bars: (**a**) 10 µm. (**b**+**c**) 1 µm.

During our studies we tested various methods for fixation, embedding and sectioning. At first, we chose the Tokuyasu CLEM approach because of its reproducibility and optimal retention of the fluorescent signal. However, cryosectioning requires special training and equipment. We tested therefore whether fluorescence of organic dyes bound to SNAP- and CLIP-tags could also be preserved in Epon epoxy sections of specimens fixed either by HPF or even chemically. Epoxy resins are the most commonly used embedding media in EM because of their superior sectioning properties. The combination of HPF and Epon embedding provides optimal preservation and contrast of cellular ultrastructures, but it destroys the fluorescence of FPs and interferes with immunolabelings. Conversely, a strong fluorescent signal was retained in labeled SOFIA mouse islets fixed by HPF followed by FS and Epon embedding (Fig. 5a). Specifically, mouse SOFIA islets were double labeled after a blocking step first with SNAP-Cell 505-Star and then with SNAP-Cell TMR-Star one day later. After one additional day in culture islets were fixed by HPF, followed by a standard FS protocol with 1% osmium tetroxide, 0.1% uranyl acetate and 1% H_2_O to increase membrane contrast^42,43^ and then Epon embedding and sectioning. Images of 505+ and TMR+ insulin SGs were acquired by SIM and correlated precisely to TEM images using as landmarks fluorescent beads^44^. A quality check of the reconstruction log-files and evaluation of the channel intensity profiles with the SIMcheck plugin^38^ confirmed the lack of reconstruction artifacts. The ultrastructural preservation was excellent, with clearly defined membranes, high contrast and the absence of ice crystal damage (Fig. 5b and c). SOFIA mouse insulin SGs lacked the characteristic halo and had a mean diameter of 265 ± 53 nm, which is close to that of rat insulin SGs in HPF samples (243 ± 73 nm, SD), while significantly smaller than that of insulin SGs (357 ± 11 nm, SE) in chemically fixed mouse islets^45^. The diameter of 2-days-old 505+ insulin SGs was 244 ± 50 nm and thereby smaller than the overall size of SGs (p__value_ < 0.05). These data are consistent with SGs undergoing changes in their size during their life-time.

### Dual Color CLEM in Epon for Investigating Insulin SG Degradation by Autophagy

**Figure 6:**
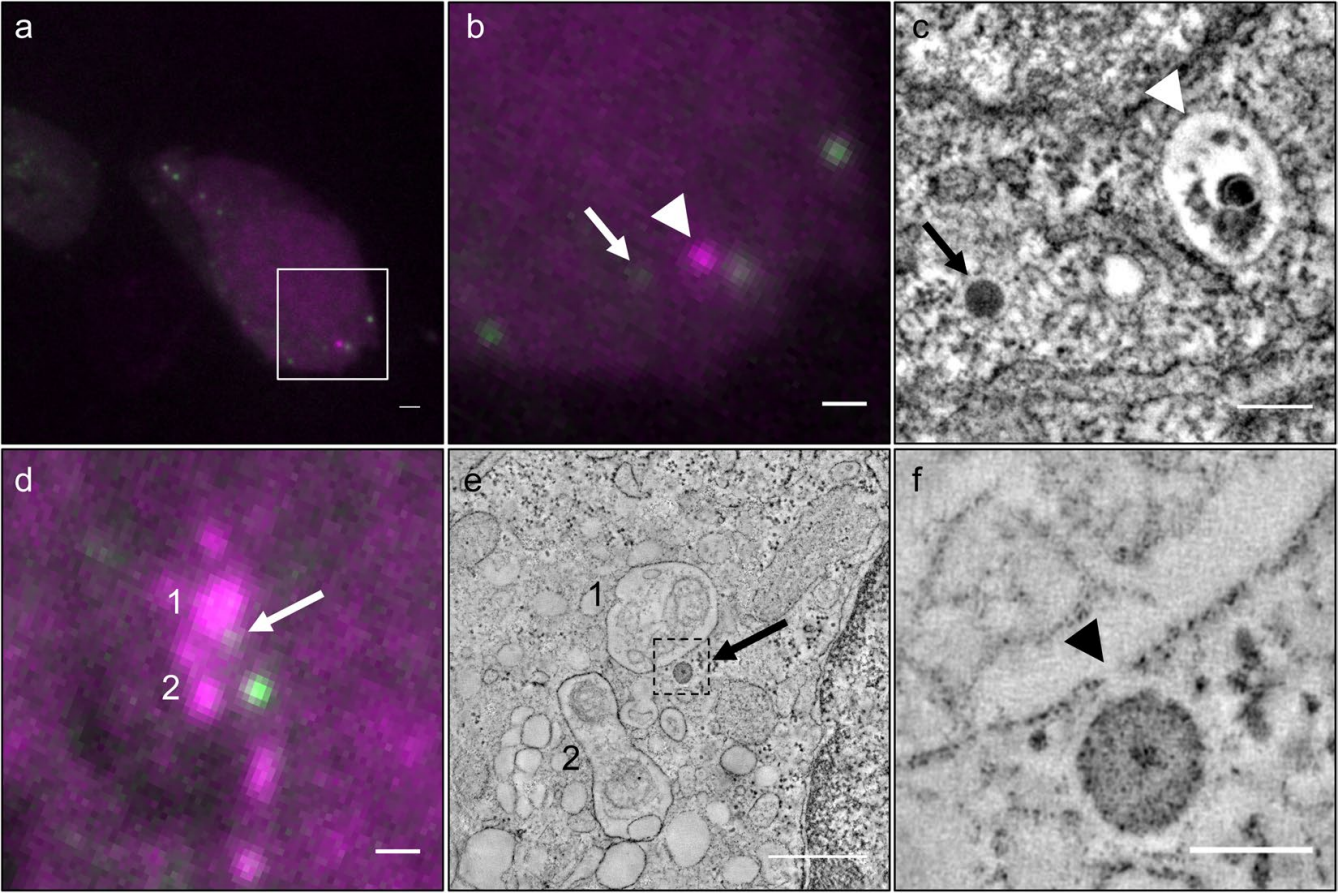
CLEM of hIns-SNAP and CLIP-LC3 in Epon after chemical fixation. (**a**) Wide-field FLM image of a 300 nm thick Epon section with several 505+ insulin SGs (green) and a TMR+ LC3 (magenta) autophagosome. (**b**) Detail of the boxed area in (**a**). The arrow and the arrowhead point to a 505+ insulin SG and to the TMR+ autophagosome. (**c**) Single slice of tomogram of area in (**b**) confirming the association of the 505+ and TMR+ signals with a SG (arrow) and an autophagosome (arrowhead), respectively. (**d**) Wide-field FLM detail image of a cell with a 505+ insulin SGs (green, arrow) and TMR+ LC3 autophagosomes (magenta, numbers). (**e**) Single slice of tomogram with the 505+ insulin SG marked in (**d**) with an arrow and the autophagosomes corresponding to TMR+ objects in (**d**) marked with numbers. (**f**): Detail of boxed area in e documenting the site of fusion (arrowhead) of the 505+ insulin SG with the TMR+ autophagosome. Scale bars: (**a**–**c**) 1 µm, (**d**+**e**) 500 nm, (**f**) 100 nm.

Insulin SGs can be disposed by autophagy^46^. To establish whether our method would be suitable for investigating this process at the ultrastructural level, we transiently co-transfected INS-1 cells with hIns-SNAP and the autophagosome marker LC3 N-terminally tagged with CLIP (CLIP-LC3). CLIP is a modified SNAP-variant that binds to substrates containing benzylcytosine instead of benzylguanine. Transfected cells were then simultaneously labeled with 10 mM SNAP-Cell 505-Star and 0.6 mM CLIP-Cell TMR-Star for 1 hour followed by 2 hours of washes and fixation with 0.25% GA and 4% PFA in sodium phosphate buffer. After block-contrasting followed by embedding in Epon both fluorescent signals were well preserved, with 505-Star appearing punctated, as typical for insulin SGs, and TMR-Star showing the cytosolic and punctated signal expected for LC3 (Fig. 6a and b). Electron tomography revealed several bonafide 505+ SGs and one LC3-TMR+ autophagosome containing an insulin SG and cytosolic material (Fig. 6c, Supplementary Movie 1). In another instance we could capture by tomography CLEM a 505+ SGs in the process of fusing with a LC3-TMR+ autophagosome (Fig. 6d–f, Supplementary Movie 2). Hence, these data extend previous morphological studies^46^ by providing ultrastructural molecular evidence for the disposal of SGs by autophagy.

### Preservation of SNAP- and CLIP-fluorescence in Epon Epoxy Resin Sections After High Pressure Freezing and Chemical Fixation

**Figure 7:**
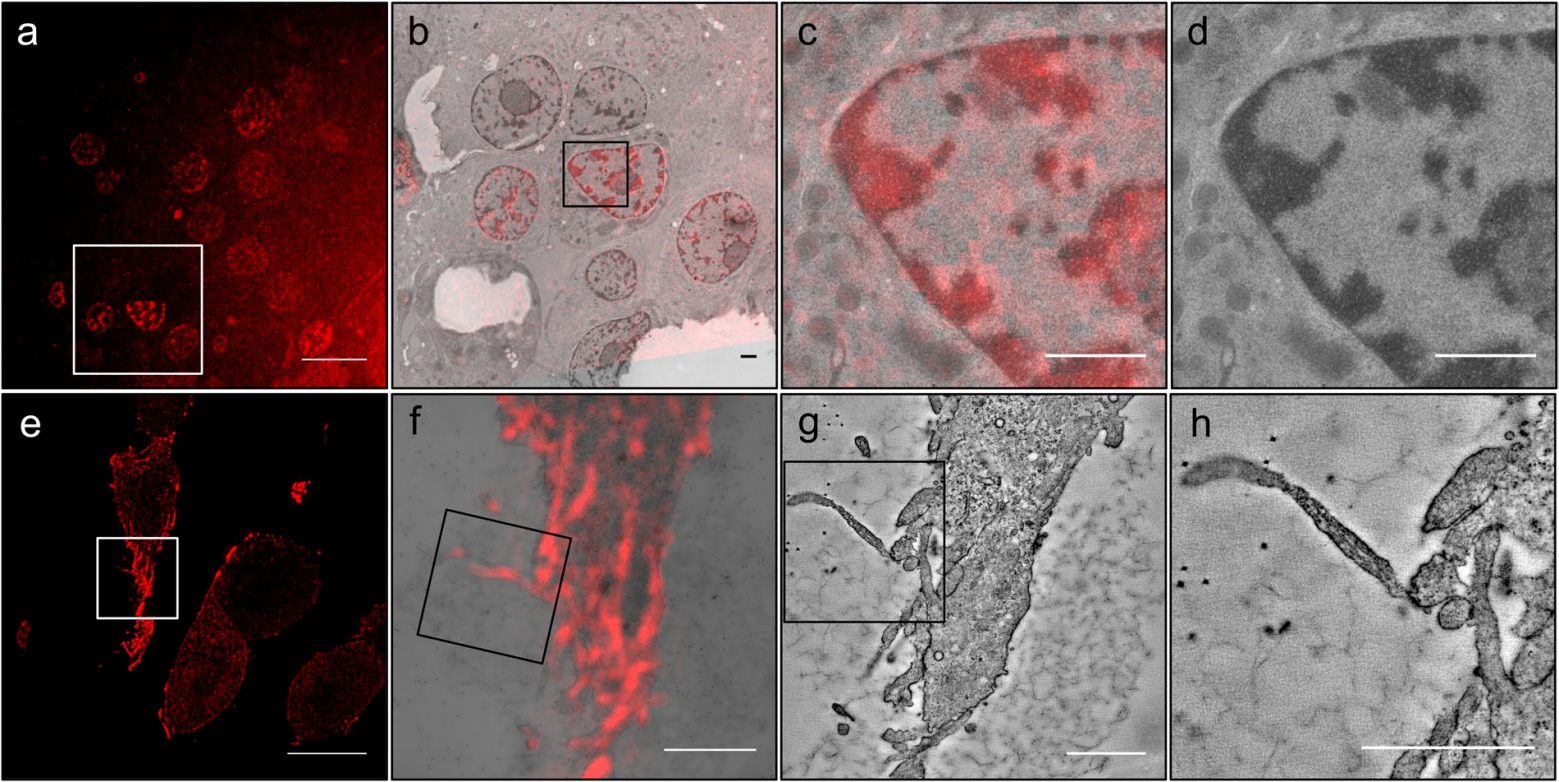
CLEM in Epon after HPF + FS and after chemical fixation. (**a**–**d**) SNAP-CCF. (**a**) Wide-field FLM image of Epon section of INS-1 cells with TMR+ ICA512-CCF-SNAP showing cytosolic and nuclear labeling. (**b**) CLEM image corresponding to the boxed area in (**a**) allowing to discriminate labeled from unlabeled cells. (**c**) CLEM detail corresponding to the boxed area in (**c**). (**d**) TEM detail showing nucleus, cell membrane and insulin SGs. (**e**–**h**) lifeAct-CLIP. (**e**) SIM image of an Epon section of INS-1 cells with TMR+ lifeAct-CLIP. (**f**) CLEM image corresponding to the boxed area in (**e**) with microvilli correlated to the fluorescent signal. (**g**) Single slice of tomogram corresponding to (**f**) with microvilli in boxed area. (**h**) Detail of boxed area in (**f** and **g**). Scale bars: (**a**) 10 µm. (**e**) 5 µm. (**b**–**d**) + (**f**–**h**) 1 µm.

The retention of fluorescence in Epon was surprising since preservation of fluorescent substrates covalently bound to tagged proteins in Epon sections has, to our knowledge, never been reported. Therefore we tested whether other fluorescently labeled SNAP- and CLIP-tagged proteins, besides insulin or LC3, could be detected in Epon sections after HPF and FS or even after chemical fixation. As a proof-of-principle INS-1 cells transiently transfected with histone protein H2B tagged with CLIP were labeled with 0.6 mM CLIP-Cell TMR-Star for 1 hour and washed for 2 hours. After HPF, FS and Epon embedding, the TMR fluorescence was retained in 100 nm thick sections (Fig. 7a). Transfected cells could be distinguished because of their fluorescent nucleus. Correlation of wide-field FLM and TEM images showed localization of H2B-CLIP to the nuclear heterochromatin, indicating the correct targeting of the reporter protein (Fig. 7a–d). Similarly, the SNAP-TMR signal was detected by CLEM in cells expressing a cytosolic portion of the transmembrane SG cargo ICA512, which translocates to the nucleus upon SG exocytosis^47,48^ (Supplementary Figure 4a). In particular, cells with different fluorescent intensities, conceivably due to different expression levels of ICA512-CCF-SNAP, could be easily distinguished. TEM imaging revealed a remarkable ultrastructural preservation without ice crystal damage and with well defined membranes (Supplementary Figure 4d, Supplementary Figure 5). Overlay of FLM and TEM images was possible using as landmarks the shapes of cells and nuclei and transfected cells could easily be identified in TEM images (Supplementary Figure 4b,c).

Chemical fixation of cell and tissue samples is still used as an important routine approach in cell biology. Unfortunately, many antigens or FPs are sensitive to strong fixatives, so that in their cases weak fixatives with 2–4% PFA are an obliged choice. Surprisingly, we found that SNAP- and CLIP-based fluorescence was retained even after strong chemical fixation with glutaraldehyde (GA) and paraformaldehyde (PFA). Notably, even in samples fixed with 2.5% GA, a clear specific fluorescence signal was detectable by wide-field FLM. In addition, use of 0.1% osmium tetroxide and 2% uranyl acetate as secondary fixatives and contrast reagents did not destroy the fluorescent signal. With this protocol we achieved a good ultrastructural preservation with strong membrane contrast and clear ultrastructural details (Fig. 7g and h, Supplementary Figures 4g and 5). In the case of INS-1 cells transfected with H2B-CLIP and labeled with CLIP-Cell TMR-Star we could clearly visualize the labeled nuclei and distinguish transfected from non-transfected cells (Supplementary Figure 4e–h). To further validate the robustness of our approach, we detected the F-actin reporter LifeAct-CLIP in Epon sections of INS-1 cells. These cells were labeled as described before and then fixed with 0.25% GA and 4% PFA in sodium phosphate buffer. After Epon embedding, 300 nm thick sections were imaged by SIM followed by post-staining and electron tomography. SIM showed a strong filamentous fluorescent signal (Fig. 7e). Electron tomography demonstrated the compartimentalization of the fluorescent signal mostly within microvilli at the cell cortex (Fig. 7f–h, Supplementary Movie 3).

## Discussion

For accurate discrimination of insulin SGs of different ages we developed a SNAP-based protocol with 2 fluorescent substrates allowing for a precise time-resolved labeling of these organelles. This protocol could be exploited for post-embedding CLEM without the need for dissociation of pancreatic islets into single cells, which negatively affects the secretory capacity of beta cells relative to clustered islet cells^49,50^. The fluorescence of the 2 substrates was strong enough for detection by SIM, which has twice the resolution of conventional FLM. Using the cell nuclei for correlation allowed for the precise superimposition in CLEM images of labeled insulin SGs within a range of few nm. Analysis of 3 experiments, each evaluating 6 age-distinct pools of SGs, confirmed the suitability of our approach for quantitative CLEM, as the number of SGs within each age-pool was highly reproducible. From these data we estimated a model showing a relatively constant number of SGs until an age of 2.7 days followed by the exponential reduction of labeled SGs. This result is in agreement with previous estimates based on pulse-chase labelings of insulin with ^3^H-leucine^51^, and indicates that insulin SG degradation is a regulated, age-dependent process. The higher abundance of SG older than 4 days found in MGBs further strengthens this conclusion. Within the recent years there have been several studies combining super resolution microscopy and EM techniques^20,52–60^. However, if image analysis was performed, CLEM was only used to confirm the subcellular localization of proteins, while quantitative analysis were exclusively performed with FLM^55,58^. In our case, instead, both EM and FLM channels were pivotal for data quantification.

A common assumption in the field of EM is that fluorescence is not retained in Epon sections. Recently, fluorescence of Alexa dyes could be preserved in epoxy resins after cell permeabilization, immunolabeling, embedding and etching of the sections allowing for STORM imaging^60^. The majority of CLEM studies rely on FPs such as GFP or mCherry. Embedding protocols prevent for various reasons the detection of FPs, which depend on their structural integrity to be fluorescent^61^. Specifically, the use of GA as primary fixative induces autofluorescence, which may shield the fluorescence of FPs. Prior to embedding samples are stained with osmium tetroxide, which is a strong oxidizer, and can therefore denaturate FPs. Uranyl acetate used as post-staining agent quenches the fluorescence of FPs^62,63^. Conversely, its use for block staining of chemically fixed samples labeled with fluorophores^62^ or in the FS cocktail for metacrylate resin embedding of samples labeled with SNAP- and Halo-substrates allows for the retention of fluorescence^20^. For the curing of Epon samples are placed at 60 °C for 24 hours, which can also lead to FP denaturation. Moreover, Epon is hydrophobic, while emission of most fluorophores requires a hydrophilic environment. Which of those factors contributes the most to the loss of fluorescence of FPs is unclear. In contrast, we show that fluorescence based on substrates bound to the SNAP-tag is preserved in samples fixed by HPF, freeze substituted with a standard cocktail for best morphological preservation containing 1% osmium tetroxide and embedded in Epon. With this approach we could reliably identify mouse beta cells in Epon sections by their SNAP-fluorescence and reevaluate the diameter of mouse insulin SGs, being was found smaller than previously reported^45^. This notion is relevant to properly estimate the number of SG exocytotic events based on capacitance measurements^36^.

Moreover, we could compare those data to the morphological analysis of age-defined insulin SGs. The results on insulin SG diameter and appearance are consistent with our previous findings in rat islets, which showed that the typical halo of insulin SGs in chemically fixed samples is not present upon fixation by HPF and represents therefore a fixation artifact^36^. Those findings however have been controversial because the morphological identification of beta cells from the other islet cell types without the help of the distinctive halo surrounding their SGs is difficult. This hurdle was clearly overcome here thanks to the unequivocal recognition of beta cells even in HPF Epon embedded samples based on the retained fluorescence of the SG Ins-SNAP reporter. The significant difference of the diameter of 2-days-old 505+ insulin SGs compared to the overall population indicates heterogeneity of insulin SG size associated with SG age.

SNAP-based fluorescence is also retained in samples treated with GA, osmium tetroxide and uranyl acetate and subsequently embedded in Epon. This phenomenon enabled us to develop a double labeling CLEM protocol for investigating the mechanisms of insulin SG degradation mediated by autophagy. Fluorescence was detected not only if associated to a protein highly concentrated in a restricted compartment, such as insulin-SNAP within the SGs or membrane-associated LC3, but also in the case of a soluble protein diffused throughout the cytoplasm and in the nucleus, such as ICA512-CCF-SNAP, hence allowing for the discrimination of transfected from non-transfected cells. The same was true for CLIP-tag based fluorescence, such as H2B-CLIP and LifeAct-CLIP. From our experiments, we conclude that fluorescent SNAP and CLIP substrates are resistant to chemical fixation with GA and treatment with osmium tetroxide and uranyl acetate followed by Epon embedding. Transfection of two proteins fused either to SNAP or CLIP followed by labeling with two substrates with different wavelengths as shown here for hIns-SNAP and CLIP-LC3 even allows for dual-color CLEM of different proteins.

HPF is regarded to be the method of choice for best ultrastructural preservation since it leads to the extremely rapid vitrification of biological samples with a thickness of up to 200 µm^64^. Combination of HPF with Epon embedding further enables the best contrast for EM. Also, Epon has superior sectioning properties compared to frozen or Lowicryl embedded samples, making it easier to obtain serial sections for standard TEM or tomography^65^. Since we demonstrated the survival of SNAP- or CLIP-based fluorescence with five different proteins in Epon sections, our approach is likely to work for a wide variety of tagged proteins. While Epon embedding after HPF and FS provides optimal ultrastructural preservation, Epon embedding after chemical fixation is the easiest, fastest and still most widely used technique for TEM. Its suitability also for CLEM is therefore especially valuable. SNAP- and CLIP-tagged reporters can be as easily expressed as FPs and, differently from the latter, can be readily exploited for “pulse/chase imaging” studies. Taken together, self-labeling protein tags can be used for robust fluorescent labeling that is retained upon different standard EM embedding and sectioning techniques. Hence, they are versatile tools for CLEM in a wide variety of cell and tissue samples.

## Methods

### Mouse and Cell Lines

The generation of SOFIA mice has been described previously^26^. The INS-1 cell line originally derived from a rat insulinoma^66,67^ was a kind gift from C. Wollheim, Geneva, Switzerland.

### Plasmids

CLIP-LC3 was generated from a GFP-hLC3 plasmid, and was a kind gift from M. Zerial (MPI-CBG, Dresden, Germany). hIns-SNAP was described earlier^26^. Lifeact-CLIP was cloned from pLifeact-mCherry, a kind gift from E. Paluch (UCL, London, United Kingdom). The construct for expression of ICA512-CCF-SNAP was generated using standard cloning techniques. The construct for H2B-CLIP was provided with the CLIP starter kit (NEB).

### Islet isolation and Cell Culture

SOFIA mouse islets were isolated according to standard procedures^68^ and cultured in RPMI 1640 (Gibco) supplemented with 10% FBS, 5.5 mM glucose, 20 mM HEPES and 100 U/ml each penicillin and streptomycin. INS-1 cells were grown in standard INS-1 media consisting of 1x RPMI 1640 medium (with L-glutamine) supplemented with 20 mM HEPES pH 7.4, 10% v/v heat-inactivated fetal bovine serum (FBS), 2 mM L-glutamine, 100 U/ml penicillin, 100 µg/ml streptomycin, 1 mM sodium pyruvate and 50 µM 2-mercaptoethanol plus 11 mM glucose.

### Transient Transfection

INS-1 cells were transiently transfected with DNA plasmids using a Laboratory Pulse Agile Electroporation System (Model PA-3000, Cyto Pulse Sciences) according to the manufacturers’ instructions.

### Age-dependent SNAP-labeling of Insulin SGs

After isolation, SOFIA mouse islets were kept in standard culture medium overnight, then treated with 20 µM BTP in standard culture medium and washed for 2 hours. Next, 10 µM 505-Star was applied for 1 hour, followed by 1 hour of washing and incubation with 2 µM BTP for 30 min followed by 30 min of washing. Finally, islets were incubated with 6 µM TMR-Star and washed again for 2 hours. Then they were either fixed or kept in culture for up to 5 days, until the desired SG age was reached.

### Continuous Protein-labeling with SNAP- and CLIP-tags

INS-1 cells were either incubated with 0.6 µM TMR-Star or 505-Star in culture medium overnight, followed by 2 hours of washes and fixation. Alternatively, cells were incubated with 6 µM TMR-Star for 1 hour, followed by washes for 2 hours and fixation.

### Sample Preparation for Tokuyasu Sectioning

For Tokuyasu cryo-sections^69,70^ labeled SOFIA mouse islets were fixed with 4% PFA in sodium phosphate buffer pH 7.4 and embedded in 12% gelatin. After incubation with 2.3 M sucrose overnight at 4 °C, samples were frozen in liquid nitrogen. Sections with a thickness of 150 nm were cut with a Leica UC6 ultramicrotome equipped with a Leica FC cryochamber (Leica Microsystems). Sections were transferred to finder grids with a carbon-coated formvar film.

### Epon embedding after chemical fixation

For preservation of SNAP- and CLIP-based fluorescence cell monolayers were fixed with a combination of 4% PFA containing either 0.25% or 2.5% GA in sodium phosphate buffer. Monolayers were postfixed/contrasted with 0.1% osmium tetroxide at 4 °C for 30 min, followed by 2% uranyl acetate at 4 °C for 20 min. Cells were then either scraped and embedded in 1% low-melting agarose or further processed for flat embedding. Cells were dehydrated with increasing concentrations of ethanol followed by embedding in EMbed812 (Electron Microscopy Sciences) with polymerization at 60 °C for 24 hours. Sections with a thickness of 100 nm or 300 nm were cut with a Leica UC6 ultramicrotome. Sections were transferred to slot grids with 3 sectors (Electron Microscopy Sciences) equipped with a thick carbon-coated formvar film. An overview of the embedding protocol is shown in Supplementary Figure 1.

### HPF, FS and Epon Embedding

Prior to the freezing procedure isolated islets or cells grown on sapphire discs were transferred into CO_2_-independent culture medium. Samples were frozen with a Leica EMpact2 high pressure freezer (Leica Microsystems). After proper freezing samples were kept in liquid nitrogen. For Epon embedding samples were freeze substituted starting with a morphology cocktail containing 1% osmium tetroxide and 0.1% uranyl acetate in acetone additionally containing 1% H_2_O for membrane contrast^42,43^. Samples were immersed in the cocktail for 5 hours at −90 °C followed by raising the temperature to 0 °C over a time course of 18 hours. Then, samples were washed twice in 100% dry acetone for 1 hour and the temperature was raised to room temperature. EMbed embedding was started after one wash with 100% dry acetone for 10 min, followed by three steps of mixtures of EMbed812 and 100% dry acetone with increasing EMbed812 concentrations for 1 hour each step. After infiltration with 100% EMbed812 overnight and another 4–6 hours in pure EMbed812 samples were transferred to specimen moulds and embedded in EMbed812 at 60 °C for 24 hours. Sections were cut as described for chemically fixed samples. An overview of the embedding protocol is shown in Supplementary Figure 1.

### Wide-field FLM for CLEM

For CLEM, Tokuyasu cryo-sections on finder grids and Epon sections on slot grids mounted between a coverslip and a microscopy slide on a drop of VectaShield antifading agent (VectorLabs). For precise correlation, Tokuyasu sections were stained with DAPI before mounting and Epon sections were sprinkled with fluorescent beads. The edges of the coverslip were sealed with nail polish. Images were taken with a Zeiss Axioplan 2 wide-field microscope (Zeiss) with a Plan-Apochromat 63x/1.4 Oil DIC objective or with a DeltaVision OMX SIM (General electric) in wide-field mode.

### Super Resolution Microscopy for CLEM

SIM images were taken with a DeltaVision OMX SIM with an Olympus Plan ApochromatN 60x oil objective with a numerical aperture of 1.42. Tokuyasu or Epon sections were imaged with immersion oil with a refractive index of 1.516. Stacks with a step-size of 125 nm and a whole z-volume of 3 µm were acquired and reconstructed with the SoftWoRx software package (SoftWoRx, Germiston, South Africa). Image quality was evaluated by checking the k0 angle shift, which is an indicator of SIM reconstruction quality and should be below 2 pixels and by checking the shift in linewidth (Daniel J. White, OMX SIM Application Specialist, Life Sciences, Cell Technologies, GE Healthcare, personal communication). Additionally, channel intensity profiles of the raw image files were assessed with the SIMcheck plugin for FIJI software^38^. For CLEM a maximum intensity projection of the stacks was created with FIJI software.

### TEM

For TEM the Tokuyasu sections were demounted from the coverslips, washed with H_2_O, contrasted on drops of methylcellulose/uranyl acetate for 10 min, looped out and dried. Epon sections were contrasted with 4% uranyl acetate and 1% lead citrate. Grids were imaged in a Tecnai 12 TEM (FEI, Hillsboro, OR) with an acceleration voltage of 100 kV. For CLEM stitched images with 8 × 8 single images at a magnification of 6,800 and with a pixel size of 1.8844194 nm were taken.

### Electron Tomography

For correlative electron tomography Epon sections with a nominal thickness of 300 nm were cut and put on slot grids with 3 sectors equipped with a formvar lm and carbon coated. Next, FLM imaging sections were post-stained with 2% uranyl acetate in 70% methanol followed by washes with methanol with decreasing concentrations and finally with H_2_O. Afterwards they were stained with 1% lead citrate. Following post-staining 15 nm colloidal gold particles were absorbed into the grids for 30 s and used as fiducial markers. Dual tilt series ranging from −64° to +64° were acquired with a Tecnai F30 electron microscope (FEI) with an acceleration voltage of 300 kV. Tomograms were reconstructed with the IMOD software package (version 4.7)^71^.

### Correlation of FLM and TEM Images

Fluorescence and TEM images were correlated using the AMIRA software (FEI) with the LandmarkWarp function. For correlation of images of Tokuyasu sections cell nuclei were used as landmarks and the corresponding points were set manually in the fluorescent and TEM images. For correlation of images of Epon sections fluorescent and electron dense beads were used as landmarks and also set manually. In the case of H2B-CLIP and ICA512-CCF-SNAP, cellular features were used as landmarks since high correlation precision was not necessary.

### Estimation of correlation accuracy

To estimate the shift between SIM and corresponding EM images ellipses were fit around SGs in SIM and their EM counterparts. The centers of those ellipses were determined by projecting the minor and major radius. The distance between the SIM and corresponding EM ellipse was measured. For each analyzed cell 1 SG close to the nucleus and 4 SGs in the periphery of the cell were measured. In total 100 SGs in 20 CLEM images were analyzed.

### Image Analysis

CLEM images were manually analyzed and the nature of correlated SGs was described depending on their age. The numbers of 505+ SGs and of all SGs within one beta cell were determined with the cell counter plugin of FIJI. Then, the percentage of labeled SGs for different ages was calculated. In 3 experiments 36–41 cells per SG age group were analyzed. We also counted the number of fluorescently labeled SGs found within MGBs for the different SG ages. Measurements of insulin SG diameters were performed in FIJI. Statistical analysis was done in Excel and Motion tracking software. ANOVA, Student’s t-test and Bayesian probability analysis were used to assess significance.

### Fit models to experimental data

The theoretical models were fitted to experimental data by mathematical tools of Motiontracking^72^ (http://motiontracking.mpi-cbg.de). The models, results of fit and uncertainty estimations^39^ are presented below:

Model 1:

1 $$y={a}\cdot \exp (-\,\frac{t}{\tau })$$

where *y* is fraction of labeled granules as function of time.

Result of fit:

$$\alpha =0.031\pm 0.0017$$

$$\tau =6.0\pm 0.8\,{\rm{days}}$$

$$\mathrm{log}\,-{\rm{likelihood}}=-\,8.82$$

Model 2:

2 $$y=\left\{\begin{array}{ll}a, & t\le {\rm{\Delta }}\\ {a}\cdot \exp (-\,\frac{(t-{\rm{\Delta }})}{\tau }), & t> {\rm{\Delta }}\end{array}\right.$$

where *y* is fraction of labeled granules as function of time.

Result of fit:

$$a=0.027\pm 0.001$$

$${\rm{\Delta }}=2.72\pm 0.43\,{\rm{days}}$$

$$\tau =2.85\pm 0.77\,{\rm{days}}$$

$$\mathrm{log}\,-{\rm{likelihood}}=-\,7.20$$

Model 3:

3 $$y=\left\{\begin{array}{ll}a\cdot \exp (-\,\frac{t}{{\tau }_{1}}), & t\le {\rm{\Delta }}\\ a\cdot \exp (-\,\frac{{\rm{\Delta }}}{{\tau }_{1}})\cdot \exp (-\,\frac{(t-{\rm{\Delta }})}{{\tau }_{2}}), & t> {\rm{\Delta }}\end{array}\right.$$

where *y* is fraction of labeled granules as function of time.

Result of fit:

$$a=0.028\pm 0.002$$

$${\rm{\Delta }}=3.0\pm 0.0004\,{\rm{days}}$$

$${\tau }_{1}=15.02\pm 10.9\,{\rm{days}}$$

$${\tau }_{2}=3.05\pm 0.76\,{\rm{days}}$$

$$\mathrm{log}\,-{\rm{likelihood}}=-\,14.2$$

### Ethical statement

All experiments involving animals were conducted according to the approved animal welfare regulations of the institutional animal ethical committee of the University Hospital Carl Gustav Carus and Faculty of Medicine of the TU Dresden, Germany (24-9168.24-1/2009-7).

## Electronic supplementary material

Supplementary Information

Supplementary video 2

Supplementary video 3

Supplementary video 4
