# Supplementary material for: A Global Approach for Quantitative Super Resolution and Electron Microscopy on Cryo and Epoxy Sections Using Self-labeling Protein Tags
Source: Sci Rep. 2017 Feb 2;7:23. doi: 10.1038/s41598-017-00033-x (PMC5428382; doi:10.1038/s41598-017-00033-x)
Supplement: Supplementary file 1 — Supplementary Information [file 41598_2017_33_MOESM1_ESM.pdf]

Supplementary Information

A Global Approach for Quantitative Super Resolution and Electron Microscopy on Cryo and Epoxy Sections Using Self-labeling Protein Tags

Andreas Müller[1, 2], Martin Neukam[1, 2], Anna Ivanova[1, 2], Anke Sönmez[1, 2], Carla Münster[1, 2], Susanne Kretschmar[3, 4], Yannis Kalaidzidis[5, 6], Thomas Kurth[3, 4], Jean-Marc Verbavatz[5, 7], Michele Solimena[1, 2, 5]

[1]Molecular Diabetology, University Hospital and Faculty of Medicine Carl Gustav Carus, TU Dresden, Dresden, Germany
[2]Paul Langerhans Institute Dresden (PLID) of the Helmholtz Center Munich at the University Hospital Carl Gustav Carus and Faculty of Medicine of the TU Dresden, Dresden, Germany
[3]Center for Regenerative Therapies Dresden (CRTD), TU Dresden, Dresden, Germany
[4]Biotechnology Center of the TU Dresden (BIOTEC), Dresden, Germany
[5]Max Planck Institute of Molecular Cell Biology and Genetics (MPI-CBG), Dresden, Germany
[6]Faculty of Bioengineering and Bioinformatics, Moscow State University, Moscow, Russia
[7]Institut Jacques Monod, Université Paris Diderot, Paris, France

*Corresponding author. Tel.: +49-351-796-366-11; E-mail: Michele.Solimena@tu-dresden.de

Supplementary Figures

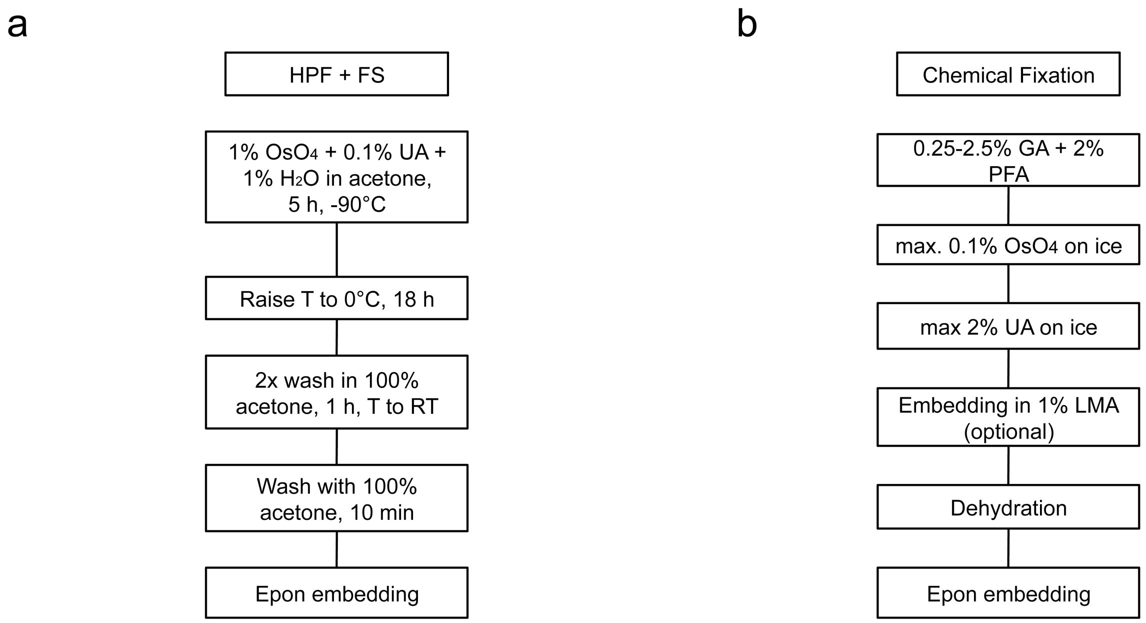

Figure S1: Protocols for Epon CLEM

a: HPF + FS protocol for preservation of CLIP- and SNAP-fluorescence.

b: Chemical fixation protocol for preservation of CLIP- and SNAP-fluorescence

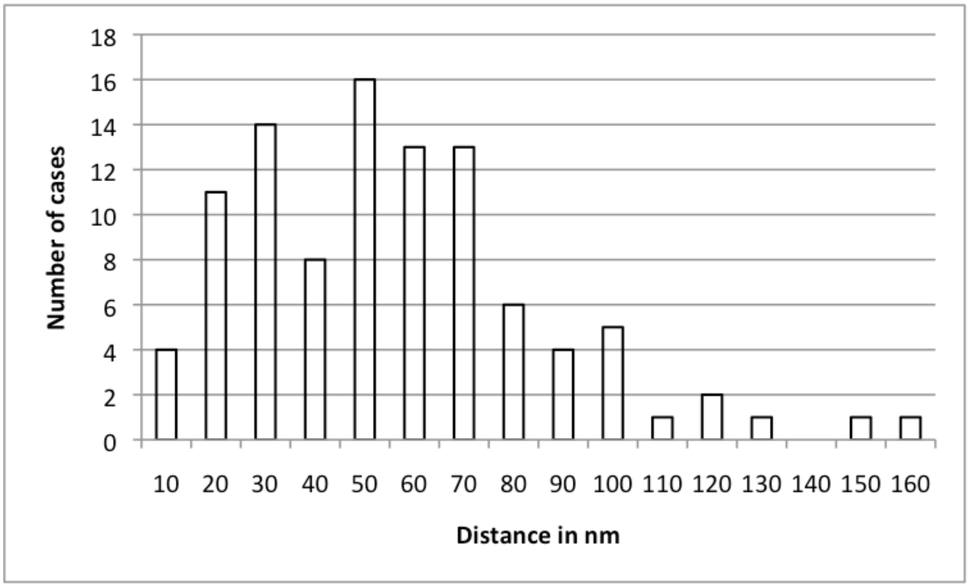

Figure S2: Shift of correlation between SIM and corresponding EM SGs

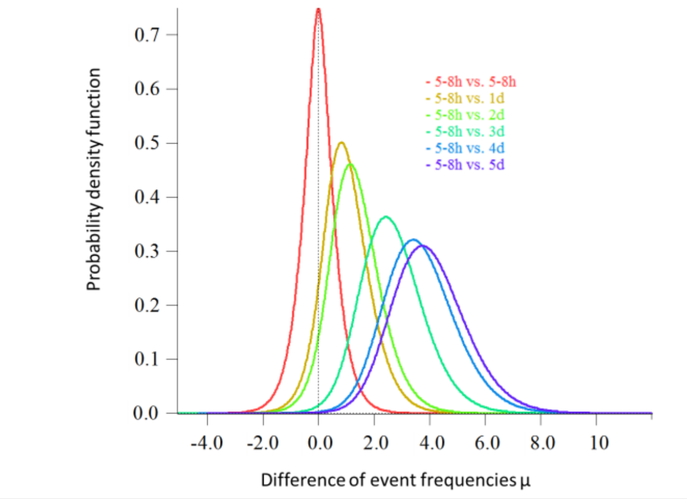

Figure S3: Probability distribution for the difference of frequencies of MGBs positive for 505+ (5-8 h compared to other SG ages)

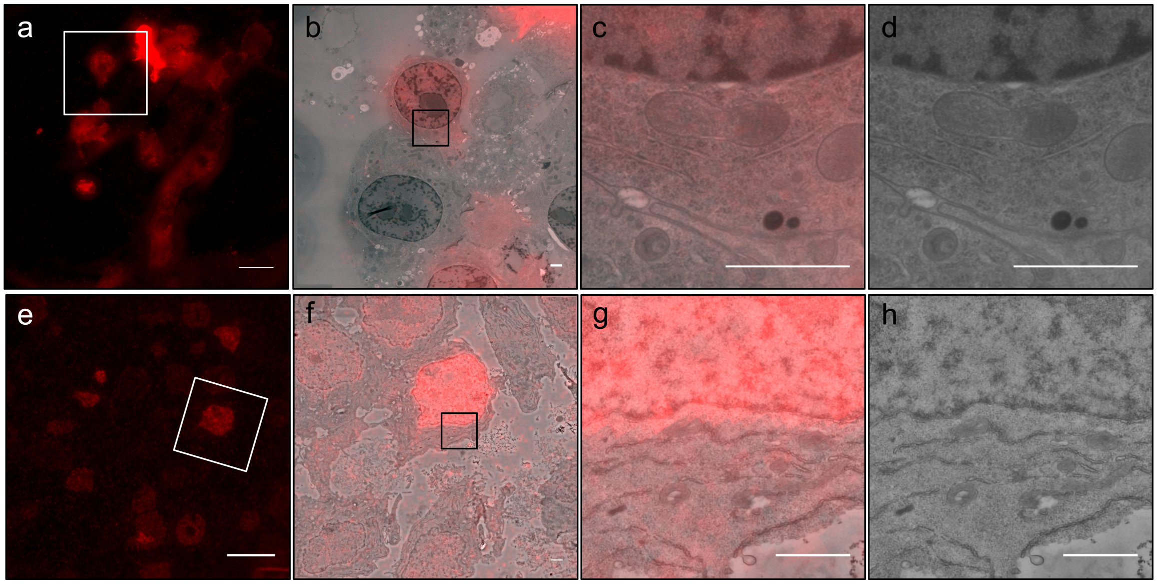

Figure S4: CLEM in Epon after HPF + FS and after chemical fixation

a-d: SNAP-CCF. a: Wide-field FLM image of Epon section of INS-1 cells with TMR+ ICA512-CCF-SNAP showing cytosolic and nuclear labeling. b: CLEM image corresponding to the boxed area in a allowing to discriminate labeled from unlabeled cells. c: CLEM detail corresponding to the boxed area in c . d: TEM detail showing nucleus, cell membrane and insulin SGs.

e-h: H2B-CLIP. e: Wide-field FLM image of an Epon section of INS-1 cells with TMR+ H2B-CLIP. f: CLEM image corresponding to the boxed area in e with TMR+ signal localized in cell nucleus. g: CLEM detail corresponding to the boxed area in f. h: TEM detail with well contrasted membranes.

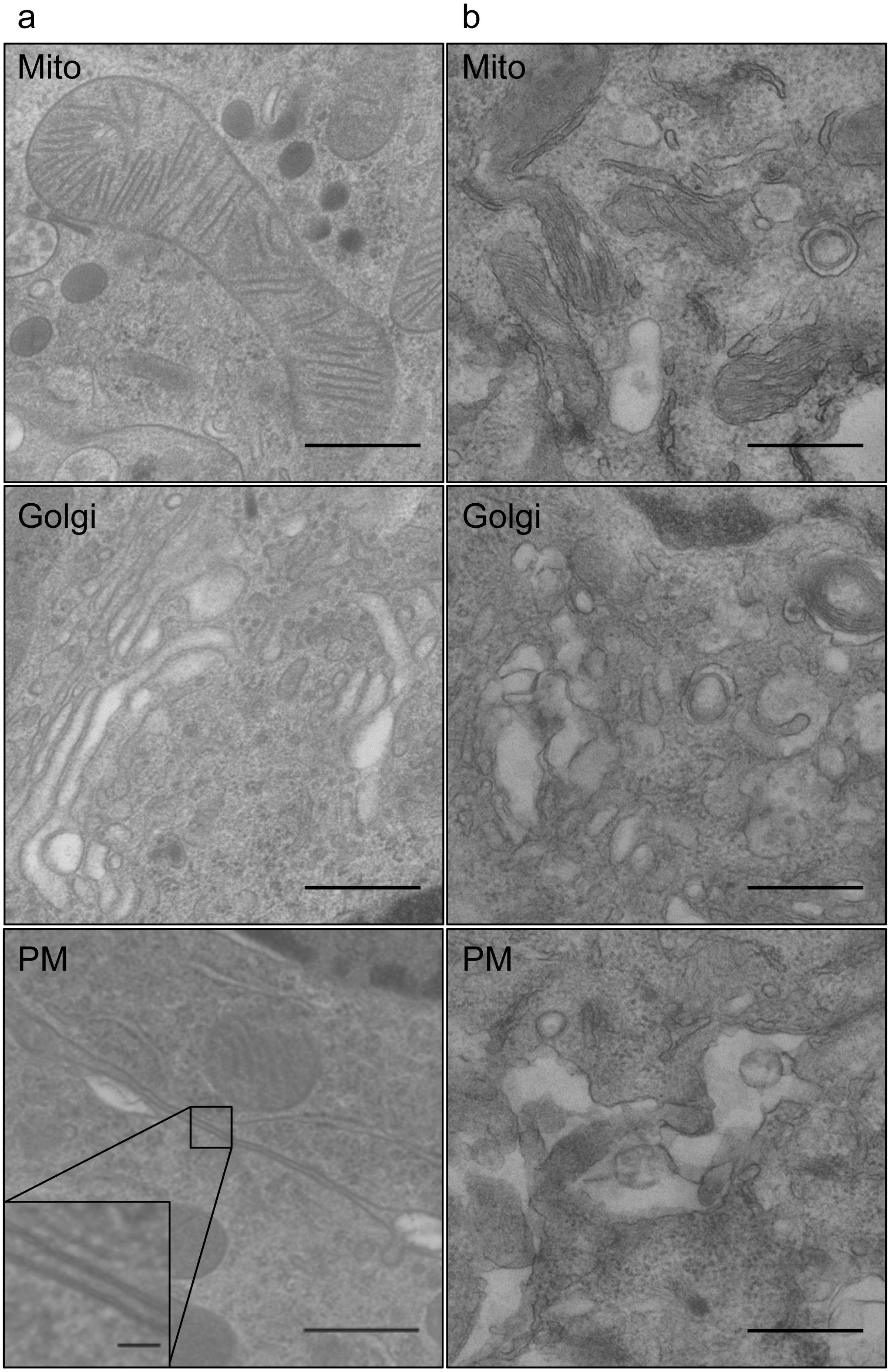
a
b
Mito
Mito
Golgi
Golgi
PM
PM

Figure S5: Examples of ultrastructural features in Epon sections used for CLEM.
a: after HPF and FS, b: after chemical fixation, Mito: Mitochondria, PM: Plasma membrane. Inset in a: PM is showing detail of lipid bilayers of 2 cell membranes. Scale bars: 500 nm, except for inset: 50 nm.

Supplementary Tables:

SG age	5-8 h	1d	2d	3d	4d	5d
5-8 h		0.383245259	0.179982068	0.337487824	0.003638637	0.000767409
1d	0.383245259		0.327640153	0.630431173	0.002168858	0.000242553
2d	0.179982068	0.327640153		0.674069059	0.067445891	0.013086835
3d	0.337487824	0.630431173	0.674069059		0.036313679	0.008038475
4d	0.003638637	0.002168858	0.067445891	0.036313679		0.02028845
5d	0.000767409	0.000242553	0.013086835	0.008038475	0.02028845	

Table S1: P-values after post-hoc t-tests

	5-8 h	1 d	2 d	3 d	4 d	5 d
Experiment 1	1	1	1	3	4	6
Experiment 2	0	2	2	4	4	4
Experiment 3	0	1	2	2	4	3

Table S2: Quantification of 505+ MGBs

	5-8h	1d	2d	3d	4d	5d
5-8h		0.11	0.063	0.0058	0.0009	0.00049
1d	0.11		0.38	0.090	0.024	0.015
2d	0.063	0.38		0.15	0.048	0.031
3d	0.0058	0.090	0.15		0.26	0.20
4d	0.0009	0.024	0.048	0.26		0.42
5d	0.00049	0.015	0.031	0.20	0.42	

Table S3: Statistical analysis of 505+ MGBs

Supplementary Methods:

A small number of observed events (MGBs containing age-defined 505+ SG) is described by Poisson statistics. The probability to observe n events, given the frequency of event λ is:

$$p(n|\lambda)=\frac{\lambda^n}{\Gamma(n+1)}e^{-\lambda}$$

If we repeat the experiment M times and observe a set of measurements $n_1,n_2,...,n_M \equiv \{n\}$, then

$$p(\{n\}|\lambda)=\prod_{i=1}^{M}\frac{\lambda^{n_i}}{\Gamma(n_i+1)}e^{-\lambda}$$

The posterior probability of λ is:

$$p(\lambda|\{n\})=\frac{p(\lambda)e^{-M\lambda}\lambda^{\sum_{i=1}^{M}n_i}}{\int_0^\infty p(\lambda)e^{-M\lambda}\lambda^{\sum_{i=1}^{M}n_i}d\lambda}=\frac{1}{Z(\{n\})}p(\lambda)e^{-M\lambda}\lambda^{\sum_{i=1}^{M}n_i} ,$$

where $p(\lambda)$ is the prior probability.

For the comparison of two conditions (e.g. 5-8h vs. 3d), we have two sets of observations $\{n_1\}$ and $\{n_2\}$with unknown frequencies λ_1 and λ_2.

Then the probability is $p(\lambda_1,\lambda_2|\{n_1\},\{n_2\})=\frac{1}{Z}p(\lambda_1)p(\lambda_2)e^{-M\lambda_1}\lambda_1^{N_1}e^{-M\lambda_2}\lambda_2^{N_2}$, where

$$N_1=\sum_{i=1}^{M}n_{1,i} \ , \ N_2=\sum_{i=1}^{M}n_{2,i}$$

Next, we build a probability distribution for the difference between unknown frequencies $\mu=\lambda_2-\lambda_1$.

For this we introduce the probability $p(\mu|\lambda_1,\lambda_2)=\delta(\lambda_2-\lambda_1-\mu)$.

Then from the probability chain rule we have

$$p(\mu,\lambda_1,\lambda_2|\{n_1\},\{n_2\})=p(\mu|\lambda_1,\lambda_2)p(\lambda_1,\lambda_2|\{n_1\},\{n_2\})$$

Following the Bayesian approach we sequentially marginalize unknown parameters λ_1 and λ_2:

$$p\left(\mu,\lambda_1 \mid \{n_1\},\{n_2\}\right)=\int_0^{\infty} p\left(\mu \mid \lambda_1,\lambda_2\right) p\left(\lambda_1,\lambda_2 \mid \{n_1\},\{n_2\}\right) d\lambda_2$$

$$=\frac{1}{Z} p\left(\lambda_2\right) p\left(\lambda_2-\mu\right) e^{-M\lambda_2} \lambda_2^{N_2} e^{-M(\lambda_2-\mu)} \left(\lambda_2-\mu\right)^{N_1}$$

, where Z is the normalization constant.

For an unbiased estimation we have chosen a improper uniform prior for unknown frequency $p(\lambda)=const$

Therefore, we get after marginalization:

$$p\left(\mu \mid \{n_1\},\{n_2\}\right)=\frac{1}{Z}\int_{\max(\mu,0)}^{\infty} e^{-M\lambda} \lambda^{N_2} e^{-M(\lambda-\mu)} \left(\lambda-\mu\right)^{N_1} d\lambda$$

$$=\frac{e^{M\mu}}{Z}\sum_{j=0}^{N_1} \frac{\Gamma\left(N_1+1\right)\left(\Gamma\left(N_2+N_1-j+1\right)-\Gamma\left(N_2+N_1-j+1,\max\left(0,2M\mu\right)\right)\right)}{\Gamma\left(N_1-j+1\right)\Gamma\left(j+1\right)}\left(-2M\mu\right)^j$$

Finally, the probability distribution is :

$$p\left(\mu \mid \{n_1\},\{n_2\}\right)=\frac{e^{M\mu}\sum_{j=0}^{N_1}\frac{\left(\Gamma\left(N_2+N_1-j+1\right)-\Gamma\left(N_2+N_1-j+1,\max\left(0,2M\mu\right)\right)\right)}{\Gamma\left(N_1-j+1\right)\Gamma\left(j+1\right)}\left(-2M\mu\right)^j}{\int_{-\infty}^{\infty} e^{M\mu}\sum_{j=0}^{N_1}\frac{\left(\Gamma\left(N_2+N_1-j+1\right)-\Gamma\left(N_2+N_1-j+1,\max\left(0,2M\mu\right)\right)\right)}{\Gamma\left(N_1-j+1\right)\Gamma\left(j+1\right)}\left(-2M\mu\right)^j d\mu}$$

We build probability distribution for difference of frequencies of MGBs positive for 505+ (Supplementary Figure 3).

Then, for every distribution we found the most probable value for the difference of frequencies, 95% confidence interval (excludes 2.5% on both side of distribution) and one-side p-value for null-hypothesis $\mu \le 0$: $p_{value}=\int_{-\infty}^{0} p\left(\mu \mid \{n_1\},\{n_2\}\right) d\mu$

	5-8h vs. 5-8h	1d vs. 5-8h	2d vs. 5-8h	3d vs. 5-8h	4d vs. 5-8h	5d vs. 5-8h
Most probable μ	0	0.83	1.14	2.43	3.41	3.74
Confidence interval (95 %)	[-1.37, 1.38]	[-0.64, 2.92]	[-0.39, 3.39]	[0.60, 5.18]	[1.36, 6.46]	[1.62, 6.88]
p-value	0.5	0.11	0.063	0.0058	0.0009	0.00049

The same calculation was done relative to day 1 and the other SG ages (not shown):

	1d vs. 1d	2d vs. 1d	3d vs. 1d	4d vs. 1d	5d vs. 1d
Most probable μ	0	0.3	1.54	3.41	3.74
Confidence interval (95 %)	[-1.28, 2.01]	[-1.85, 2.59]	[-0.79, 4.35]	[0.01, 5.63]	[0.28, 6.04]
p-value	0.5	0.37	0.090	0.024	0.015

Supplementary Movies

Movie S1: Tomogram of LC3-CLIP TMR+ and hIns-SNAP 505+ INS-1 cell

Scale bar: 500 nm

Movie S2: Tomogram of LC3-CLIP TMR+ and hIns-SNAP 505+ INS-1 cell with SG fusing with autophagosome

Scale bar: 500 nm

Movie S3: Tomogram of LifeAct-CLIP TMR+ INS-1 cell

Scale bar: 500 nm
